# Rare BRAF mutations in pancreatic neuroendocrine tumors may predict response to RAF and MEK inhibition

**DOI:** 10.1371/journal.pone.0217399

**Published:** 2019-06-03

**Authors:** Amy Allen, Alice Can Ran Qin, Nitya Raj, Jiawan Wang, Sharmeen Uddin, Zhan Yao, Laura Tang, Paul A. Meyers, Barry S. Taylor, Michael F. Berger, Rona Yaeger, Diane Reidy-Lagunes, Christine A. Pratilas

**Affiliations:** 1 Department of Oncology, Sidney Kimmel Comprehensive Cancer Center at Johns Hopkins, Johns Hopkins University School of Medicine, Baltimore, Maryland, United States of America; 2 Department of Medicine, Memorial Sloan Kettering Cancer Center, New York, New York, United States of America; 3 Molecular Pharmacology Program, Memorial Sloan Kettering Cancer Center, New York, New York, United States of America; 4 Department of Pathology, Memorial Sloan Kettering Cancer Center, New York, New York, United States of America; 5 Department of Pediatrics, Memorial Sloan Kettering Cancer Center, New York, New York, United States of America; 6 Marie-Josee and Henry R. Kravis Center for Molecular Oncology, Memorial Sloan Kettering Cancer Center, New York, New York, United States of America; Rutgers University, UNITED STATES

## Abstract

The clinical significance of *BRAF* alterations in well-differentiated (WD) metastatic pancreatic neuroendocrine tumor (panNET) is unknown, but *BRAF*-mutated panNET could represent a subset characterized by an identifiable and clinically actionable driver. Following the identification of two patients with WD metastatic panNET whose tumors harbored *BRAF* mutations, we queried the MSK-IMPACT series of 80 patients with WD metastatic panNET for additional mutations in *BRAF*, and in other genes involved in RAS/ RTK/ PI3K signaling pathways. *BRAF* mutations were identified in six samples (7.5%): two tumors harbored V600E mutations, one tumor each expressed K601E, T599K, and T310I mutations, and one tumor expressed both G596D and E451K BRAF. Few additional actionable driver alterations were identified. To determine the ERK activating capability of four *BRAF* mutations not previously characterized, mutant constructs were tested in model systems. Biochemical characterization of *BRAF* mutations revealed both high and low activity mutants. Engineered cells expressing BRAF K601E and V600E were used for *in vitro* drug testing of RAF and MEK inhibitors currently in clinical use. BRAF K601E demonstrated reduced sensitivity to dabrafenib compared to BRAF V600E, but the combination of RAF plus MEK inhibition was effective in cells expressing this mutation. Herein, we describe the clinical course of a patient with *BRAF* K601E and a patient with *BRAF* V600E WD metastatic panNET, and the identification of four mutations in *BRAF* not previously characterized. The combined clinical and biochemical data support a potential role for RAF and MEK inhibitors, or a combination of these, in a selected panNET population.

## Introduction

Pancreatic neuroendocrine tumors (panNET) are an uncommon and heterogeneous group of cancers, representing 1–2% of all cancers originating in the pancreas. While many of these tumors exhibit slow-growing and indolent behavior, most patients present with metastatic disease, and ultimately succumb to this cancer. Recent research efforts to understand the genomic landscape of this disease have identified changes in chromatin remodeling genes and in elements of the mTOR pathway in a subset of well-differentiated (WD) panNET, but few clinically actionable driver alterations [1, 2].

Following the identification of an index case of a patient with a *BRAF*-mutant WD panNET, we evaluated the frequency of *BRAF* alterations in a large clinical series of patients with WD panNET. *BRAF* alterations are known to commonly occur in other neural-crest derived tumors, including melanoma, and in high-grade neuroendocrine cancers. Previous studies have not identified *BRAF* alterations in WD panNET, but instead have consisted of a small number of cases and focused largely on the V600 hotspot in *BRAF*; these prior studies have identified *BRAF* alterations in poorly differentiated neuroendocrine carcinomas as well as WD NET originating in the colon and rectum [3, 4]. As *BRAF* alterations would represent a potentially targetable driver in WD panNET that may be sensitive to selective RAF and MEK inhibitors, in a disease without other targetable alterations, we queried the incidence and spectrum of *BRAF* alterations in a cohort of WD panNET sequenced at our institution.

BRAF is a serine/threonine kinase in the classical mitogen-activated protein kinase cascade; activation of BRAF leads to MEK and consequently ERK activation, which in turn regulates cell function in a variety of ways including activation of transcriptional programs and regulation of proliferation. Two classes of *BRAF* alterations that lead to its constitutive activation have been identified: (1) *BRAF* V600 mutations, which generate mutant proteins that can signal as monomers in the absence of RAS activation and (2) non-V600 activating mutations or fusions, which lead to RAF dimerization independent of RAS activation [5, 6].

Given the importance of lesions in the ERK pathway as drivers of transformation, there have been extensive efforts to develop drugs that inhibit components of the pathway. Selective allosteric inhibitors of MEK have activity against *BRAF* V600-mutated tumors and a subset of those with *RAS* mutations [7–13]. Trametinib (Novartis) is the first of this class to gain FDA approval, either as a single agent or in combination with a RAF inhibitor for *BRAF* V600 mutant melanoma [14–16]. Selective ATP-competitive RAF inhibitors have also been developed [17]. Two of these (vemurafenib, Genentech/ Roche; and dabrafenib, Novartis) have shown clinical activity and are approved for treatment of patients with BRAF-mutated melanoma [18–20]. RAF inhibitors effectively inhibit ERK signaling only in tumors in which the pathway is driven by mutant V600 BRAF. In normal cells and other tumors, these drugs activate the pathway [5, 21–23]. In tumors with mutant V600 *BRAF*, in which RAS activation is feedback suppressed by the constitutive, high ERK pathway activity, mutant BRAF signals predominantly as monomers, and is therefore sensitive to current RAF inhibitors.

To study the genomic drivers of advanced WD panNET, we performed next-generation sequencing (NGS) of tumor tissue in the routine practice setting using an institutional matched tumor-normal NGS platform (MSK-IMPACT). We identified few alterations in known driver genes in this metastatic WD panNET cohort. In six patients (6 of 80, 7.5%), however, potential driver alterations in *BRAF* were identified, and included both V600E mutations and non-V600 mutations. With the understanding of the potential driver role of BRAF in tumors, and our novel finding of *BRAF* alterations in WD metastatic panNET, we studied these cases further. Herein, we highlight two cases of patients with *BRAF*-mutated WD metastatic panNET treated with targeted therapies. We expressed the BRAF mutants identified in our series and evaluated their effects on downstream signaling and sensitivity to small molecule inhibitors of RAF. We focused on *BRAF* K601E, as it is the most frequently reported in cancers among the non-V600 *BRAF* alterations that we identified, and has been previously reported to activate the ERK pathway [6, 24]. We studied the *in vitro* response of this particular mutation to both RAF and MEK inhibitors, and to these drugs in combination, in order to understand the potential clinical utility of these agents in patients with non-V600 mutations in BRAF.

## Materials and methods

### Cell lines, antibodies and reagents

A375, SKBR3 and NIH-3T3 cells were purchased from the American Type Culture Collection between 2015 and 2018 and grown in the recommended medium. NT-3 cells were obtained from Dr. Jorg Schraeder at the University Medical Center Hamburg-Eppendorf, maintained in RPMI+Glutamax supplemented with 10% fetal bovine serum (FBS), fibroblast growth factor (FGF) and epidermal growth factor (EGF). NT-3 cells were direct sequenced to confirm the absence of mutations in *HRAS*, *KRAS* and *NRAS*. BON cells were acquired from Dr. Mark Hellmich at the University of Texas Medical Branch, and grown in DMEM:F12 medium with 10% FBS. SIG-M5 and JVM3 cells were obtained from Dr. Omar Abdel-Wahab at Memorial Sloan Kettering Cancer Center (MSKCC) and were maintained in RPMI medium with 10% FBS. 3T3 cell lines were engineered to stably express BRAF mutations using a doxycycline-inducible system. Antibodies against ERK, pERK, MEK, pMEK, GAPDH and V5 were obtained from Cell Signaling (Beverly, MA); BRAF antibody was obtained from Santa Cruz Biotechnology (Dallas, TX). EGF and FGF were acquired from Peprotech (Rocky Hill, NJ). PLX4032 (vemurafenib), GSK2118436 (dabrafenib), GSK1120212 (trametinib), and lapatinib were purchased from SelleckChem (Houston, TX). Drugs for *in vitro* studies were dissolved in dimethyl sulfoxide (DMSO) to yield 10mM or 1mM stock solutions, and stored at -20°C.

### Generation of mutants and transfections

The pcDNA3.1-BRAF-V5 was constructed as previously described [5, 25]. Mutations were induced by using site-directed mutagenesis kit (Stratagene) and confirmed by Sanger sequencing. For novel BRAF mutants, primer sets were designed as described in the Methods in S1 Methods, and used in PCR. Cells were seeded in 60 mm dishes and transfected the following day using Lipofectamine 2000 (Invitrogen) according to the manufacturer’s instructions. The ratio of DNA to lipofectamine was 1 μg DNA/3 μl lipofectamine.

### Immunoblotting

Cells were disrupted on ice for 30 min in 1% NP-40 lysis buffer. Protein concentration was determined with BCA reagent (Pierce Chemical Co., Rockford, IL). Equal amounts of protein were separated by SDS-PAGE, transferred to membranes, immunoblotted with specific primary and secondary antibodies, and detected by chemiluminescence with ECL detection reagents.

### Cell proliferation assays

Proliferation of cell lines was measured at 72 hrs following exposure to drug or to DMSO. Cells were seeded at a density of 6*10^4^ cells/ well in 6-well dishes for 18–24 hours and then treated with the indicated drugs for 72 hrs, in triplicate for each dose shown. Cells were collected by trypsinization and counted using a BioRad TC20 Automated Cell Counter. Average number of viable cells at 72 hrs is expressed as percent relative to DMSO control for each of the cell lines.

### IMPACT testing and generation of oncoprint figures

Genomic profiling of tumor samples was performed as previously described [26, 27]. Patients whose tumors were sequenced by IMPACT were consented and enrolled on Memorial Sloan Kettering Cancer Center institutional review board (IRB) approved clinical trial, MSKCC 12–245. Patients gave written and verbal consent.

MSKCC clinical dataset and publicly available data sets were queried for the genes of interest using cBioPortal.org [28, 29].

## Results

### Clinical MSK-IMPACT testing of panNET

**Fig 1:**
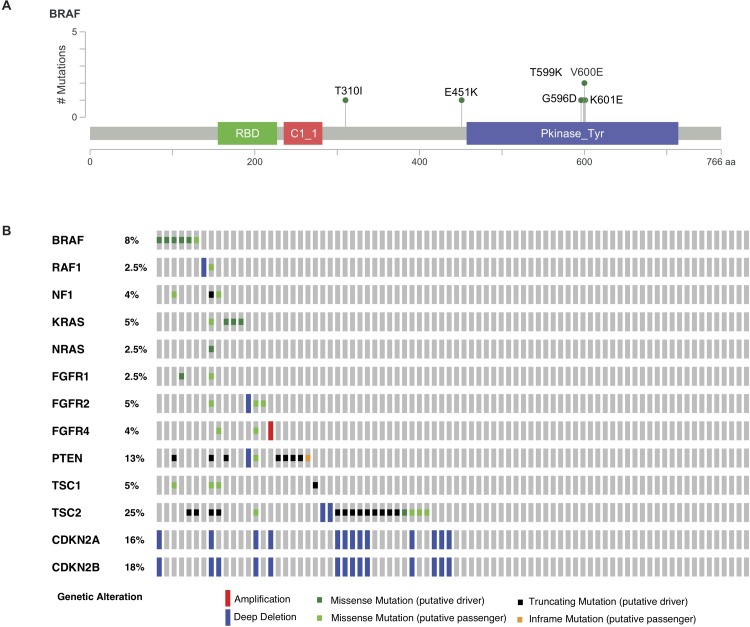
Identification of BRAF mutations in the MSK-IMPACT clinical series of panNET. A. Lollipop plot demonstrating BRAF mutations identified by MSK-IMPACT in our series of panNET. B. Identification of RTK/ RAS/ RAF/ PI3K pathway mutations in MSKCC clinical series of panNET (n = 80). First two columns/ samples represent tumors with BRAF V600E. (Complete list of genes included in query is included in S1 Methods. Genes for which no mutations were identified are not shown in the plot.)

Following the introduction of the MSK-IMPACT NGS panel into the routine clinic setting in 2014, we identified a total of 80 patients with WD metastatic panNET who had NGS performed on their tumor tissue (up to March 2017). Among the 80 patients, mutations in *BRAF* were identified in six cases (two V600E, one K601E, one T599K, one T310I, and one with *BRAF* G596D and E451K mutations, 7.5%, (Fig 1A). In other tumor types, such as melanoma, colorectal, and lung cancer, activating *BRAF* mutations occur mostly in a non-overlapping distribution with other genes that converge on activation of ERK signaling pathways (i.e., *KRAS*, *NRAS*, *NF1*, *EGFR*). We therefore queried the MSK clinical series for other mutations in RTK, RAS and PI3K signaling pathway genes that occur in these tumors (Fig 1B). In this cohort of tumors, the median somatic mutational burden was 2.95 mutations/Mb (range 0 to 201.76; compared to 3.9 for all tumor types in the MSKCC-IMPACT series) [30]. Consistent with prior observations from both whole genome and exome sequencing in locally advanced and metastatic WD panNET [2], other notable mutations identified in the tumors of these 80 patients included mutations in the chromatin remodeling genes *MEN1* (56%), *DAXX* (40%), *ATRX* (25%), and the mTOR pathway genes *TSC2* (25%) and *PTEN* (13%), data is shown in S1 Fig and [31].

We then attempted to address the overall frequency of rare, non-V600E *BRAF* mutations in human cancer. Over one hundred different mutations and alterations in *BRAF* have been described; the majority (95%) of which represent missense coding mutations, with very few in-frame deletions and truncating mutations comprising the remainder (www.cBioPortal.org) [28, 29]. Of a total of over 2200 alterations in *BRAF* described from the 159 cancer genomic studies represented in cBioPortal, V600E mutations clearly represent the vast majority, with mutations at position K601 included within the top five next most commonly mutated codons (together with D594, N581, G466 and G469, ranging from 28–67 mutations reported, compared to > 1000 for V600, data shown in S2 Fig. Upon review of MSKCC patient tumors sequenced by IMPACT, *BRAF* mutations were identified in 622 samples (6%), and of those, 19 were K601E (3%) [30]. The remaining mutations in *BRAF* identified in our panNET series (T310I, T599K, E451K and G596D) are not previously described, with the exception of G596D, which occurs in four cases as cited in the Catalog of Somatic Mutations In Cancer (https://cancer.sanger.ac.uk/cosmic). Clinical histories of those patients with recurrent mutations in *BRAF* are therefore described.

### Clinical history of patient #1

The patient was an 11-year-old girl at the time of diagnosis, who underwent pancreaticoduodenectomy for a localized pancreatic tumor; pathology revealed WD panNET. She developed recurrence within the year, biopsy-proven, and was treated with cytotoxic therapy with good response. Approximately three years later, CT scan showed marked increase in disease (bilateral peri-celiac lymphadenopathy as well as mesenteric and retroperitoneal lymphadenopathy). She began treatment with temozolomide and bevacizumab with disease stabilization for approximately one and a half years, at which time therapy was stopped due to thrombocytopenia. Her disease progressed over the ensuing years despite somatostatin analog therapy, a second debulking surgery, treatment with targeted agents (sunitinib and everolimus), and peptide receptor radiotherapy (PRRT).

Approximately ten years after her original diagnosis, a biopsy of an enlarging supraclavicular lymph node was performed and WD intermediate-grade disease (Ki-67 proliferative index 10%) was confirmed; a commercial multi-gene NGS panel was used and a K601E *BRAF* mutation was the only alteration identified. Based on this finding, the patient began treatment with trametinib. However, before initiating therapy with trametinib, a left cervical lymph node was excised, with pathology again demonstrating WD, intermediate-grade disease (Ki-67 proliferative index 20%). Unfortunately, only weeks later, the disease progressed and as a result, dabrafenib was added to trametinib. She tolerated the combination well without further adverse side effects. Imaging performed seven weeks following the initiation of combination dabrafenib and trametinib therapy showed new marked colitis, suggestion of bowel ischemia, and massive disease progression. Trametinib and dabrafenib were discontinued, and the patient died of progressive disease six days later.

Of note, MSK-IMPACT testing was done retrospectively after the patient’s death, on both the supraclavicular lymph node metastasis, which had been found to harbor *BRAF* K601E, and on the left cervical lymph node excised immediately prior to start of treatment with trametinib. *BRAF* K601E was confirmed in the former, but not detected in the latter sample, as shown in the table in S1 Table.

### Clinical history of patient #2

The patient was a 37-year-old woman initially diagnosed with a presumed signet ring cell adenocarcinoma at an outside hospital. She was initiated on therapy locally with FOLFOX (folinic acid, 5-fluorouracil, oxaliplatin) and bevacizumab. She presented to MSK for a second opinion after several months on chemotherapy, at which time, endoscopic ultrasound-guided biopsy of three lesions in the body of the pancreas was done, and pathology review reclassified the tumor as metastatic WD low-grade panNET (Ki-67 proliferative index 2%). The patient was transitioned to treatment with 5-fluorouracil, leucovorin, and bevacizumab. Upon progression, biopsy was performed in the liver and reconfirmed metastatic WD, low-grade panNET (Ki-67 proliferative index <3%). Genomic profiling of the liver metastasis was performed using the MSK-IMPACT platform and identified a *BRAF* V600E mutation.

The patient then initiated chemotherapy with capecitabine and temozolomide for 10 months, and subsequently the disease progressed. She then enrolled in a basket clinical trial of vemurafenib [20]; as part of this clinical trial, a repeat biopsy of the liver lesion was performed before treatment and sequencing using MSK-IMPACT confirmed the presence of a *BRAF* V600E mutation. The patient achieved stable disease with some minor shrinkage noted on imaging for approximately seven and a half months. Upon disease progression, the patient’s tumor became hormone-secreting (adrenocorticotropin). She underwent hepatic arterial embolization at which time a liver biopsy was taken, and demonstrated WD, but now high-grade disease (Ki-67 proliferative index 60%); MSK-IMPACT again confirmed the presence of a *BRAF* V600E mutation. She subsequently received a cycle of chemotherapy with carboplatin and etoposide before succumbing to her disease.

### Novel alterations in BRAF identified in panNET samples include both high and low activity BRAF mutations

**Fig 2:**
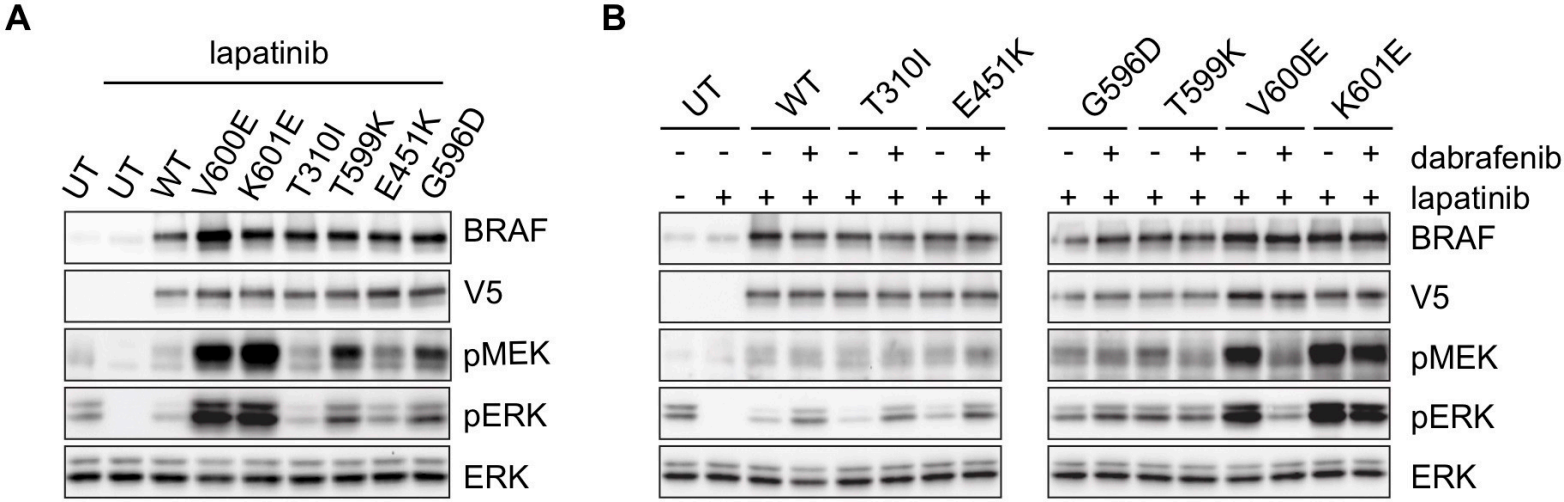
ERK activating ability and sensitivity to dabrafenib of BRAF V600E, K601E, and four BRAF mutations not previously characterized. A-B. V5-tagged BRAF mutants (V600E, K601E, T310I, T599K, E451K and G596D) were expressed in SkBr3 cells overnight and treated with lapatinib (1uM for 1 hour) (A), and then followed by treatment with DMSO or dabrafenib (200nM) for 1 hour (B). UT = untransfected. Expression and/or phosphorylation of the indicated proteins were assessed by immunoblot.

In addition to the V600E mutation, we identified several non-V600 alterations in *BRAF*. We therefore generated mutant constructs of these mutations in order to assess their effect on signaling and sensitivity to dabrafenib. PanNET preclinical models are scarce, and available panNET models are unfortunately not useful for testing the biochemical effects of BRAF mutations, as two of the three available cell lines harbor activating mutations in NRAS and KRAS as shown in data in S3 Fig [32, 33]. Only NT-3 cells, a more recently established cell line [34], lacks an oncogenic mutation in one of the RAS genes, and we therefore studied the effect of introducing the BRAF mutations into these cells. As the optimal evaluation of signaling induced by the mutant constructs occurs in the context of low endogenous RAS activity, we used SkBr3 cells that harbor high-level *HER2* amplification and in which RAS activity can be modulated by treatment with lapatinib, as previously shown [6, 35]. Of the mutations tested, BRAF V600E and K601E clearly demonstrated the most profound induction of ERK signaling (Fig 2A). T599K and G596D also induced phospho-ERK (pERK) levels above wild-type (WT) BRAF, albeit less than that induced by V600E. Introduction of T310I and E451K demonstrated only slightly increased levels of pERK comparable to that elicited by introduction of WT BRAF. We then tested the ability of dabrafenib to inhibit ERK signaling downstream of these mutations. Dabrafenib inhibited pMEK and pERK downstream of transfected K601E (although less than V600E), but not G596D and T599K (Fig 2B). The two remaining mutants exhibited paradoxical activation of ERK with dabrafenib treatment. These data suggest that K601E, T599K, and G596D are activating BRAF mutants, while T310I and E451K are “low activity” BRAF mutants [24].

### Biochemical and biological evidence for RAF and MEK inhibitor use in pancreatic NET cells harboring K601E mutation in BRAF

**Fig 3:**
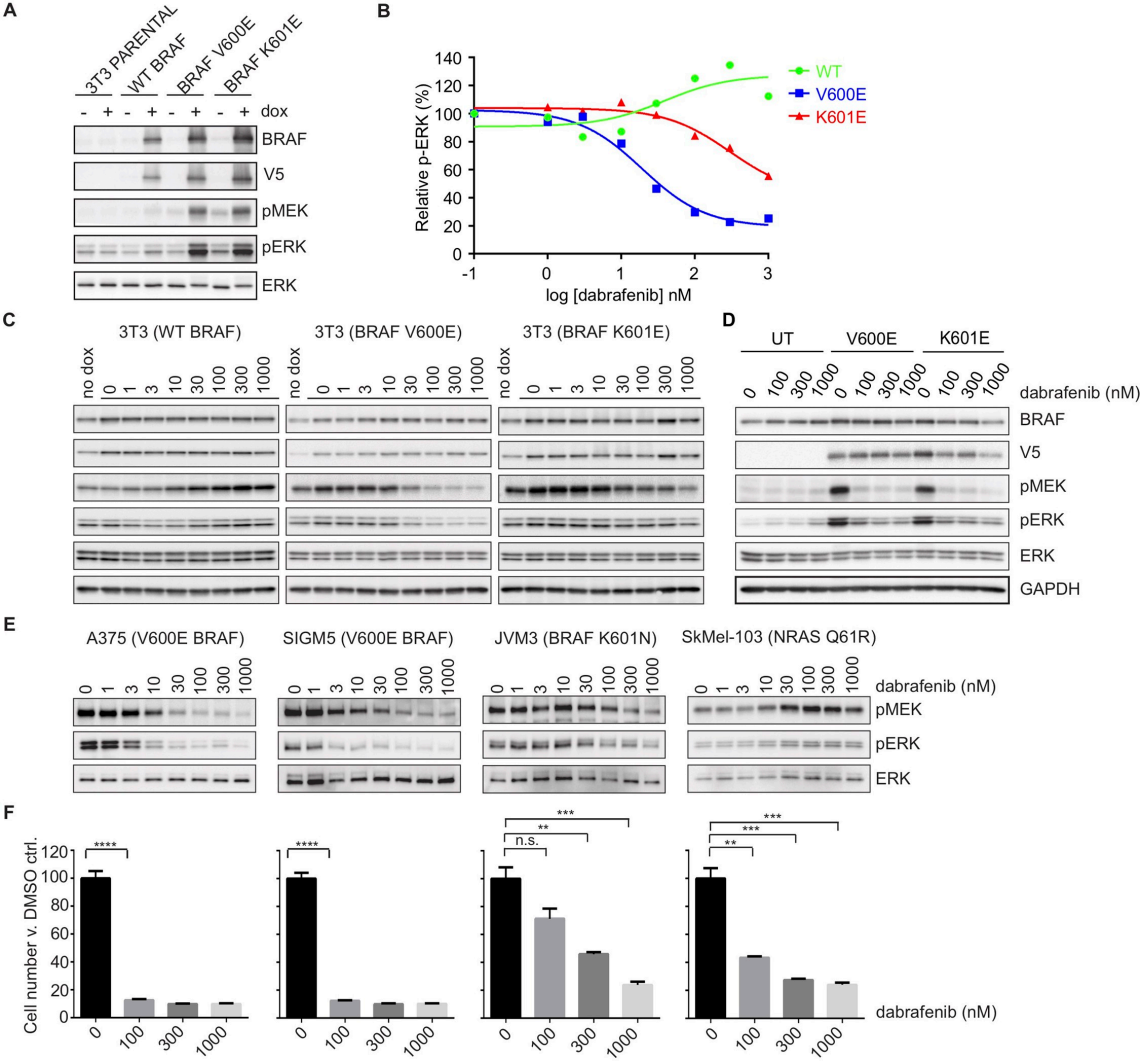
BRAF K601E induces similar levels of phospho-ERK, but has reduced sensitivity to dabrafenib compared to BRAF V600E. A-C. NIH-3T3 cells were stably transfected with doxycycline-inducible BRAF mutants (WT, V600E and K601E). A. Cells were plated in the presence (+) or absence (-) of doxycycline. B-C. Cells were plated in the presence of doxycycline (unless otherwise indicated), followed by treatment with dabrafenib (dose range as shown) for 1 hour (quantitation (B)) is shown for phospho-ERK immunoblots (C). D. NT-3 cells were transiently transfected with BRAF mutants (V600E and K601E) and treated with various doses of dabrafenib (as indicated) for 1 hour. E. Cell lines as shown were treated with dabrafenib, over a dose range, for 1 hour. UT = untransfected. In A–E, expression and/or phosphorylation of the indicated proteins were assessed by immunoblot. F. Cell lines, as in E, were plated in 6-well dishes and treated with the indicated doses of dabrafenib for 72 hrs, in triplicate for each dose shown. Average number of viable cells at 72 hrs is expressed relative to DMSO control for each cell line. Data represent mean ± SEM; ****, P<0.0001; ***, P<0.001; **, P<0.01; *, P<0.05; n.s. = no significance; unpaired Student’s t-test.

We then focused on the clinical implications of the *BRAF* K601E alteration, as this is the most common (and only recurrent) mutation in human cancer of the non-V600 alterations that we identified, and its sensitivity to current RAF inhibitors is unknown. We expressed K601E BRAF in 3T3 cells using a doxycycline-inducible system and observed that activation of MEK and ERK was similar in intensity to activation under the expression of BRAF V600E (Fig 3A). Next we tested the *in vitro* effects of two clinically available RAF inhibitors, vemurafenib and dabrafenib, and the MEK inhibitor trametinib, in cells engineered to express BRAF V600E and BRAF K601E, and in those endogenously expressing mutations in these amino acids. The available leukemia cell lines JVM3 (K601N BRAF) and SIG-M5 (V600E BRAF) were used for comparison of these two mutations since no pancreatic neuroendocrine cell lines exist that express these mutations (and attempts to culture the patient’s cancer cells were unsuccessful). We found that dabrafenib inhibited MEK and ERK phosphorylation in cells expressing K601-mutant BRAF, albeit at higher doses (300-1000nM) than what is required for V600E BRAF expressing cells (30-100nM) (Fig 3). This was true for both 3T3 cells stably expressing the mutant forms of BRAF (Fig 3B, quantitation of Fig 3C, immunoblots) and in leukemia cells with endogenous expression of these two BRAF mutants (SIGM5 and JVM3, Fig 3E). We also expressed BRAF V600E and K601E in a WD panNET cell line, NT-3 [34], in which we confirmed the absence of mutations in *HRAS*, *NRAS* and *KRAS*, and found that dabrafenib inhibited MEK and ERK phosphorylation to a similar extent in the context of both mutations (Fig 3D). These findings are in contrast to the paradoxical activation of MEK and ERK that is seen with dabrafenib treatment of cells expressing WT BRAF (Fig 3C) and those with activating mutations in *RAS* (SkMel-103, Fig 3E and 3F).

**Fig 4:**
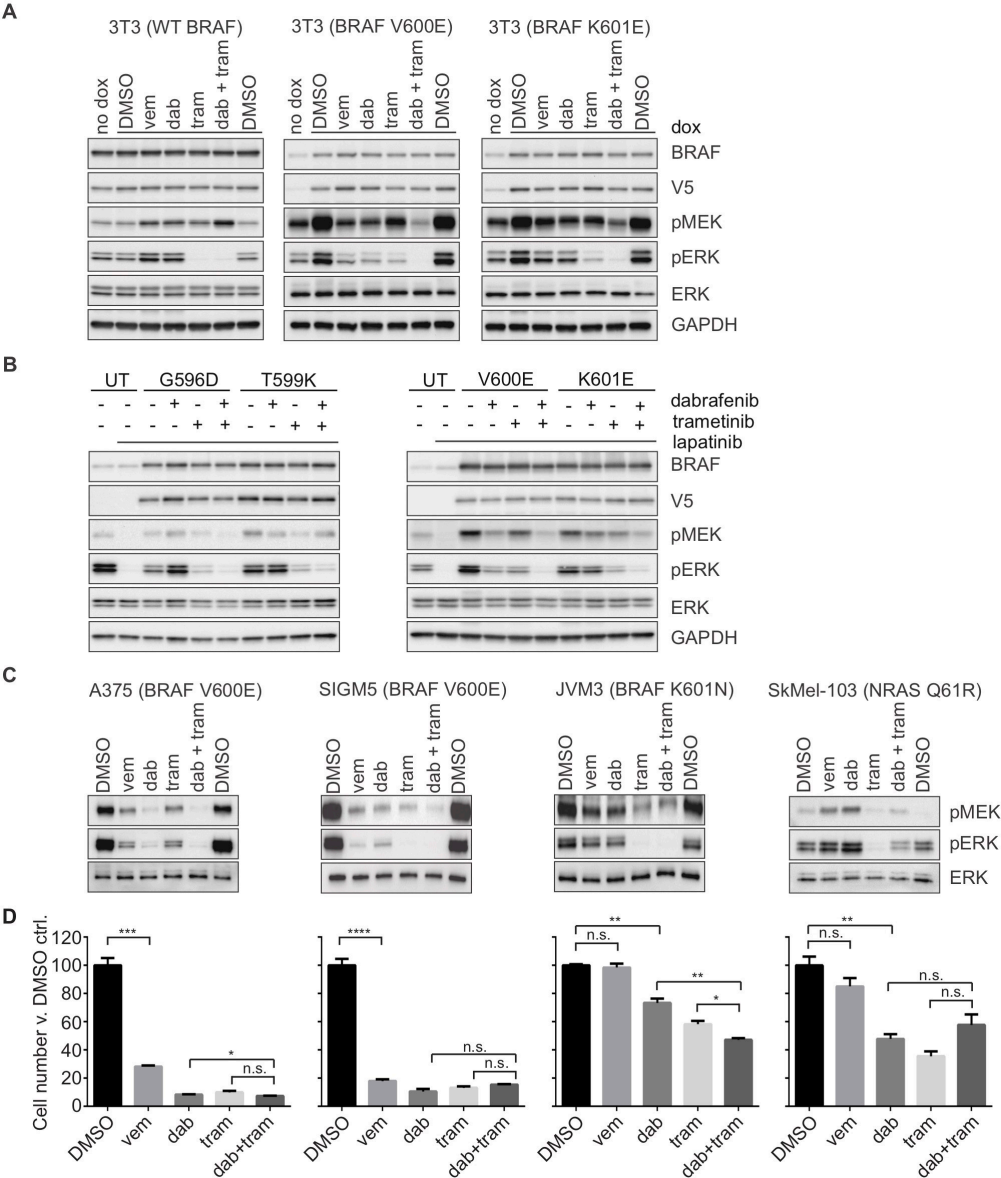
RAF plus MEK inhibition more potently inhibits phospho-ERK and proliferation in cells expressing BRAF K601 mutations than RAF inhibition alone. NIH-3T3 cells stably transfected with doxycycline-inducible BRAF mutants (WT, V600E and K601E) were plated in the presence of doxycycline (unless otherwise indicated) (A); SKBr3 cells, transiently transfected with V5-tagged BRAF mutants, pre-treated with lapatinib, as shown (B); and cell lines (C) were treated with vemurafenib (1000nM), dabrafenib (100nM, A and C, and 200nM, B), trametinib (20nM), or the combination of dabrafenib (100nM, A and C, 200nM, B) plus trametinib (20nM), as indicated, for 1 hour. (B). UT = untransfected. Expression and/or phosphorylation of the indicated proteins were assessed by immunoblot. D. Cell lines, as in C, were plated in 6-well dishes and the indicated drugs for 72 hrs, in triplicate for each dose shown. Average number of viable cells at 72 hrs is expressed relative to DMSO control for each cell line. Data represent mean ± SEM; ****, P<0.0001; ***, P<0.001; **, P<0.01; *, P<0.05; n.s. = no significance; unpaired Student’s t-test.

The combination of RAF and MEK inhibitors produces more durable inhibition of ERK signaling and more potent effects on tumor growth inhibition in models of BRAF V600E [36]. Since MEK inhibitor treatment causes release of MEK from ERK-dependent feedback and leads to reactivation of MEK in cells other than those with V600E BRAF [25], it was not clear what effects on signaling would occur when these two agents were administered in combination to non-V600E BRAF mutant-expressing cells. We therefore investigated whether more complete inhibition of ERK signaling could be achieved in BRAF mutant-expressing cells by administration of RAF and MEK inhibitors in combination. In fact, dabrafenib plus trametinib produced potent inhibition of ERK phosphorylation at one hour of treatment, and cell proliferation at three days, both in the V600E and in the K601 mutant model, but not in the WT BRAF model, nor in a model expressing an activating Q61R mutation in *NRAS* (Fig 4A–4D**)**. Cells expressing G596D and T599K BRAF did not exhibit inhibition of pERK in response to dabrafenib, but the MEK inhibitor alone, or in combination with dabrafenib, elicited a potent inhibition of pERK (Fig 4B).

### K601E BRAF requires dimerization for activity

**Fig 5:**
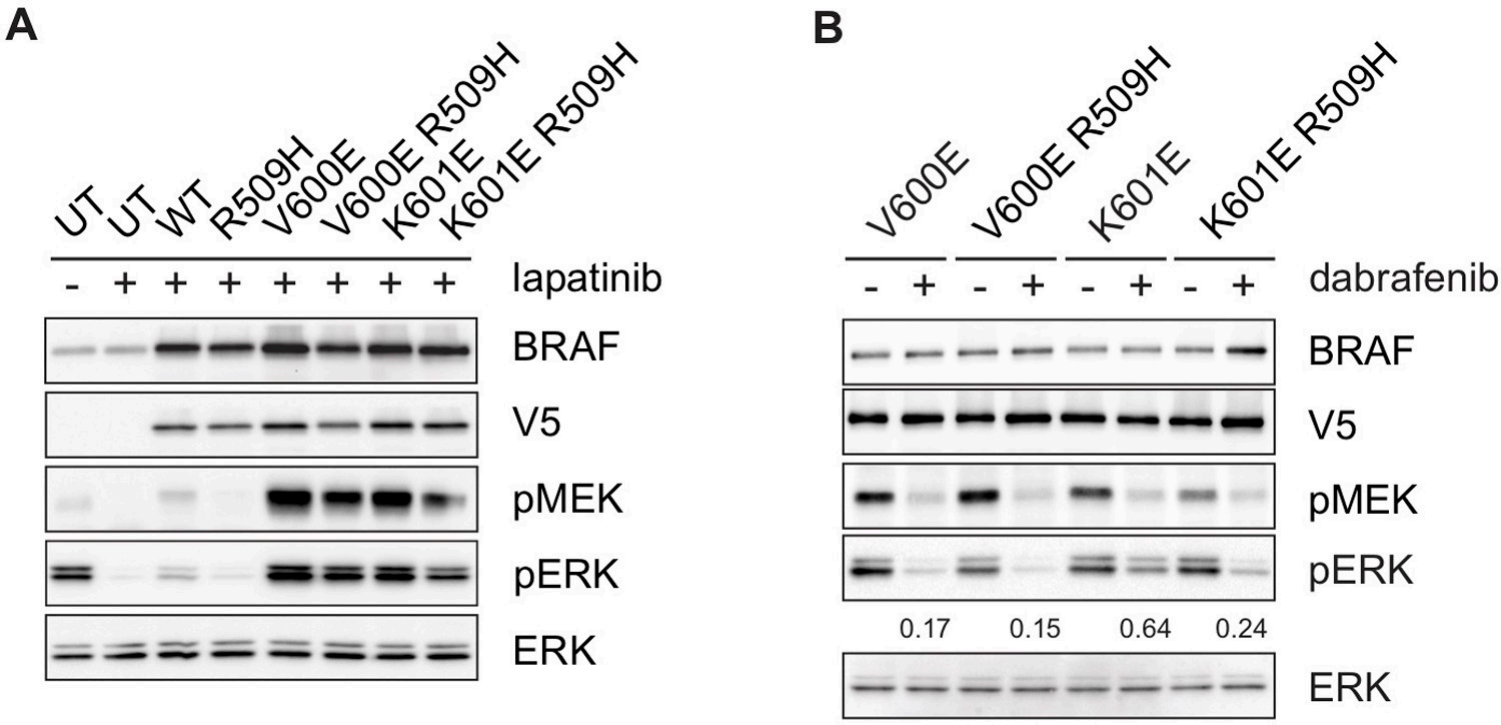
BRAF K601E requires dimerization for activity. A-B. V5-tagged BRAF mutants (WT/ R509H/ V600E/ V600E R509H/ K601E/ K601E R509H) were expressed in SKBR3 cells overnight, followed by lapatinib treatment (1uM for 1 hour) only (A), then followed by treatment with DMSO or dabrafenib (200nM) for 1 hour (B). UT = untransfected. Expression and/or phosphorylation of the indicated proteins were assessed by immunoblot. Phospho-ERK levels for dabrafenib treatment are given as a percent of pERK expression in the untreated sample, for each individual mutation, normalized for V5 expression.

BRAF molecules other than V600 mutants (wild-type and most other mutants) rely on the formation of RAF dimers in order to activate MEK and ERK signaling [6, 37]. These molecules are not susceptible to currently available RAF inhibitors, as the RAF inhibitor causes a RAS-dependent activation of the RAF dimer and results in activation of MEK and ERK signaling. Since MEK and ERK phosphorylation induced by the K601E mutant is partially inhibited in response to (somewhat higher concentrations of) dabrafenib, we asked whether it in fact relies on dimer formation for its activity. Although RAS-independent, it is previously shown that this mutant requires dimerization for its activity [6]. We used a tool mutation, R509H, that disrupts dimer formation in each of these mutants [37], and then tested the ability of dabrafenib to inhibit ERK signaling when each of these molecules was expressed in an isogenic system. As expected, both V600E and V600E R509H produce elevated levels of pERK that are reduced by the addition of dabrafenib. K601E produces a similarly elevated level of pERK, which is also partially inhibited by dabrafenib. Expression of K601E R509H, however, reduced pERK expression compared to K601E, and the degree to which this mutant was inhibited by dabrafenib was greater, confirming that K601E is also dimerization dependent (Fig 5A and 5B), therefore limiting the clinical utility of currently available RAF inhibitors as single agents in tumors in which this mutation is found.

## Discussion

In our large series of WD metastatic panNET, we find that *BRAF* alterations are recurrent and potentially targetable. *BRAF* alterations included V600E and, in some cases, non-V600 activating mutations. Our analysis of signaling in cells with *BRAF* K601E mutation suggests that both tumors with V600E *BRAF* and tumors with some non-V600 activating *BRAF* mutations may be sensitive to combined RAF and MEK inhibition.

Several dozen mutations in the gene encoding BRAF kinase have been identified in human tumors. The majority of these mutations lie within the kinase domain, and of these, the majority has been characterized as high-kinase activity mutants. The most common *BRAF* mutation, V600E, is well described, and small molecules that inhibit mutant BRAF kinase are now approved for clinical use [18, 38]. Other mutations in *BRAF*, even those within the kinase domain, occur with dramatically reduced frequency and therefore the biology of these mutations, and the implications for use of drugs targeting BRAF in these tumors, are not well established. Among them, so-called high-activity mutants have kinase activity greater than the wild-type counterpart and signal to ERK through MEK. The K601E mutant falls into this class, has been shown to result in elevated levels of phospho-MEK and phospho-ERK in tumors in which it is found, and has been described in melanoma and in colon and thyroid cancers. To our knowledge, ours is the first report of the K601E *BRAF* mutation in a WD panNET. We also identified four novel mutations in *BRAF*, T599K and G596D, which confer high kinase activity, and T310I and E451K, which are consistent with low-activity mutations in *BRAF*. These mutants were expressed in NT-3 panNET cells and in SKBr3 HER2-expressing breast cancer cells, to determine their effect on downstream signaling, and BRAF K601E RAF inhibitor sensitivity was further studied in 3T3 cells. We acknowledge that our current study is partially limited by the use of both panNET and non-panNET models in order to conduct the transfection experiments using novel BRAF mutations. Although the exclusive use of panNET cell lines would have been ideal, this option was precluded by the finding that at least two of the commonly used panNET cell lines harbor oncogenic mutations in RAS [32, 33], and therefore represent unsuitable model systems in which to study the biochemistry of BRAF mutations and their signaling. Therefore, we emphasized the use of the SKBr3—lapatinib model that is well-suited for this purpose due to the ability to modulate RAS signaling by inhibiting HER2 [6, 35]. This system allows the study of BRAF biochemistry without the confounding role of RAS-induced dimerization. Knowledge of the ability of novel mutations to activate the RAF-MEK-ERK signaling pathway, and their response to RAF inhibition, will be useful for other patients in whom these mutations are identified.

Only a small number of published studies have described mutations in *BRAF* in neuroendocrine tumors. One study analyzed 40 NET of the gastroenteropancreatic system and found one mutation in *BRAF* (V600E) in a single tumor in the cohort and reported activated levels of phospho-ERK in nearly all tumors in the cohort [39]. Another study, which performed whole exome sequencing in ten panNET found no mutations in *BRAF* or other RAS pathway activating genes [1], as shown in data in S4 Fig. In a more recent study, the authors conducted whole genome sequencing on a series of 102 primary panNET [2]. This study elegantly classified panNET into five different mutational signatures, including a novel class characterized by germline mutations in *MUTYH*. It is interesting to note that in this study, no somatic mutations in *BRAF* were identified. These two series of panNET included both locally advanced and metastatic tumors [1, 2], whereas our cohort included only patients with advanced, progressive, metastatic disease. The more advanced disease state of our cohort overall could potentially account for the identification of *BRAF* mutations in our series, but not in others, as mutations in *BRAF* are associated with aggressive biology in other gastrointestinal cancers.

We have identified a subset of WD metastatic panNET with potentially actionable driver alterations. Our preclinical data suggest that this subset of tumors should be sensitive to RAF and/or MEK inhibitors. Our patient with *BRAF* V600E panNET benefitted from disease control with vemurafenib; but unfortunately, our patient with *BRAF* K601E panNET had continued disease progression with dabrafenib plus trametinib. Our data suggest that this may have been multifactorial: ERK is only modestly inhibited with the RAF inhibitor alone in *BRAF* K601 mutant tumors and, in this patient a starting dose of 50% of the recommended dose was given based on her declining overall status. Secondly, an additional tumor site biopsied from this patient at a later date, distant from the tumor known to harbor *BRAF* K601E, was not found to express this mutation, suggesting that tumor heterogeneity and/ or polyclonality may have contributed to the lack of overall response. It is well recognized that tumors are characterized by biological and genomic heterogeneity, which increases in complexity particularly in advanced and metastatic disease states. This genomic evolution is particularly important in a disease such as panNET that often progresses indolently over years. In further genomic and clinical analysis of the MSK series of WD metastatic panNET [31], we have illustrated the concept of tumor heterogeneity in panNET, as well as genomic progression as a result of time and treatment. Genomic and biological evolution, therefore, could have certainly contributed to the lack of response seen in our patient. In addition, this patient also began the targeted therapy, at a time when her disease burden was high, after progression on multiple standard-of-care options, potentially limiting the ability to obtain a meaningful clinical response so near the end of her life. There are data that BRAF V600E tumors that respond to targeted therapies and later progress can de-differentiate on progression, so it is interesting that the tumor transformed to a high grade after vemurafenib treatment. Finally, the responses to BRAF-targeted therapeutic approaches are variable across tumor types, with limited success of these agents in colorectal cancer, for example. While the small number of patients treated makes it challenging to conclude definitively, our data suggest that BRAF mutants seen in panNET may represent an opportunity for a histology-agnostic targeted therapeutic approach, and that patients with metastatic panNET should be screened for *BRAF* alterations and enrolled in matched clinical trials, where appropriate.

## Supporting information

S1 Methods Description of query used to create oncoprints in Figs 1B and S1. List of primer sets used for generation of BRAF mutant constructs used.
(DOCX)

S1 Fig Oncoprint of BRAF mutant cases from MSK clinical IMPACT series. Series, (n = 6), shown to demonstrate overlap with genes from *Raj et al*. (*MSKCC clinical series of panNET*, [31]).
(PDF)

S2 Fig Incidence of non-V600 alterations in BRAF in cBioPortal (159 cancer studies, 2282 alterations in BRAF). **A.** Lollipop plot of all missense, in-frame, truncating, and other mutations in BRAF, n = 2282; V600 represents the most commonly altered codon, and the y-axis is set at 100 to enhance visualization of next five most common codons altered in cancer. **B.** Distribution of V600, K601, D594, G466, G469 and N581 mutations among cases in which BRAF is altered (n = 2282). References [28–30].
(PDF)

S3 Fig BRAF mutant expression in BON cells. V5-tagged BRAF mutants (as indicated) were expressed in BON (NRAS Q61R) cells [32] overnight and followed by treatment with dabrafenib, over a dose range, for 1 hour. UT = untransfected. Expression and/or phosphorylation of the indicated proteins were assessed by immunoblot.
(PDF)

S4 Fig Query of panNET data in cBioPortal.org. Genes encoding members of the RTK/ RAS/ RAF/ PI3K pathways in which mutations were found in our series (*BRAF RAF1 NF1 KRAS NRAS FGFR1 FGFR2 FGFR4 PTEN TSC1 TSC2 CDKN2A CDKN2B*, see Fig 1B) were used to query two published panNET data series using cBioPortal.org: *Jiao et al*, *Science 2011*, (BRAF mutations in 0 out of 10) [1]; and *Scarpa et al*, *Nature 2017*, (BRAF mutations in 0 of 98) [2].
(PDF)

S1 Table IMPACT analysis of the patient’s tumors over her disease course. Her 2013 tumor recurrence, and only one of her two resected recurrent sites of disease in 2014, harbored the BRAF K601E mutation. The other lymph node metastasis, resected, and from which the cell line was attempted, was confirmed to have no evidence of BRAF K601E (0 of 861 reads).
(PDF)
