# Supplementary material for: Rare BRAF mutations in pancreatic neuroendocrine tumors may predict response to RAF and MEK inhibition
Source: PLoS One. 2019 Jun 3;14(6):e0217399. doi: 10.1371/journal.pone.0217399 (PMC6546234; doi:10.1371/journal.pone.0217399)
Supplement: S1 Methods — List of primer sets used for generation of BRAF mutant constructs used. (DOCX) [file pone.0217399.s001.docx]

**Supplemental Methods**

**S1 Methods**

1. Mutational landscape of panNET. Oncoprint (Figures 1B and S1) and lollipop plot (Fig 1A and S2) were generated using cBioPortal.org. The MSKCC clinical series of patients with panNET (n=80) was identified, and then manually queried for the presence of genes involved in RTK/ RAS/ RAF/ PI3K signaling pathways (*BRAF, ARAF, RAF1, NF1, HRAS, KRAS, NRAS, EGFR, ERBB2, ERBB3, ERBB4, KIT, FGFR1, FGFR2, FGFR3, FGFR4, IGF1R, INSR, PDGFRA, PDGFRB, FLT1, KDR, FLT3, FLT4, MET, NTRK1, NTRK2, NTRK3, DDR1, PIK3CA, PIK3CB, PIK3CG, PIK3CD, PIK3R1, AKT1, AKT2, AKT3, PTEN, MTOR, TSC1, TSC2, CDKN2A, CDKN2B).* Genes in which no putative driver alterations (amplification, deletion, missense mutation or truncating mutation) were identified were eliminated from further study. Genes for which driver alterations or alterations with presumed loss of tumor suppressor function were identified in at least one sample are shown in Figure 1B. Six BRAF mutant cases were then used to determine overlap between BRAF mutation and genes identified as recurrently mutated [1]*.* 159 cancer studies in the cBioPortal public database were queried for the presence of BRAF mutations, and the frequency of V600, as well as the next five most commonly occurring sites of mutation, were calculated relative to all alterations in BRAF. References [2-4].
2. For generation of BRAF mutant constructs, the following primer sets were used in PCR:

- BRAF_T310I_F TCCTTAGCAGAGATTGCCCTAACATCTGGA
- BRAF_T310I_R GATGTTAGGGCAATCTCTGCTAAGGACGCC
- BRAF_T599K_FTTGGTCTAGCTAAAGTGAAATCTCGATGGAGTGG
- BRAF_T599K_R CGAGATTTCACTTTAGCTAGACCAAAATCACC
- BRAF_E451K_F AGTGATGATTGGAAGATTCCTGATGGGCAG
- BRAF_E451K_R CATCAGGAATCTTCCAATCATCACTCGAGTCC
- BRAF_G596D_F TAGGTGATTTTGATCTAGCTACAGTGAAATCT
- BRAF_G596D_R ACTGTAGCTAGATCAAAATCACCTA

**Supplemental References**

1. Raj N, Shah R, Stadler Z, Mukherjee S, Chou J, Untch BR, et al. Real-Time Genomic Characterization of Metastatic Pancreatic Neuroendocrine Tumors Has Prognostic Implications and Identifies Potential Germline Actionability. JCO Precision Oncology. 2018. doi: DOI: 10.1200/PO.17.00267.

2. Gao J, Aksoy BA, Dogrusoz U, Dresdner G, Gross B, Sumer SO, et al. Integrative analysis of complex cancer genomics and clinical profiles using the cBioPortal. Sci Signal. 2013;6(269):pl1. Epub 2013/04/04. doi: 10.1126/scisignal.2004088. PubMed PMID: 23550210; PubMed Central PMCID:PMC4160307.

3. Cerami E, Gao J, Dogrusoz U, Gross BE, Sumer SO, Aksoy BA, et al. The cBio cancer genomics portal: an open platform for exploring multidimensional cancer genomics data. Cancer Discov. 2012;2(5):401-4. Epub 2012/05/17. doi: 10.1158/2159-8290.CD-12-0095. PubMed PMID: 22588877; PubMed Central PMCID:PMC3956037.

4. Zehir A, Benayed R, Shah RH, Syed A, Middha S, Kim HR, et al. Mutational landscape of metastatic cancer revealed from prospective clinical sequencing of 10,000 patients. Nat Med. 2017. doi: 10.1038/nm.4333. PubMed PMID: 28481359.
