# Supplementary figures and images for: Rare BRAF mutations in pancreatic neuroendocrine tumors may predict response to RAF and MEK inhibition
Source: PLoS One. 2019 Jun 3;14(6):e0217399. doi: 10.1371/journal.pone.0217399 (PMC6546234; doi:10.1371/journal.pone.0217399)

## S1 Figure

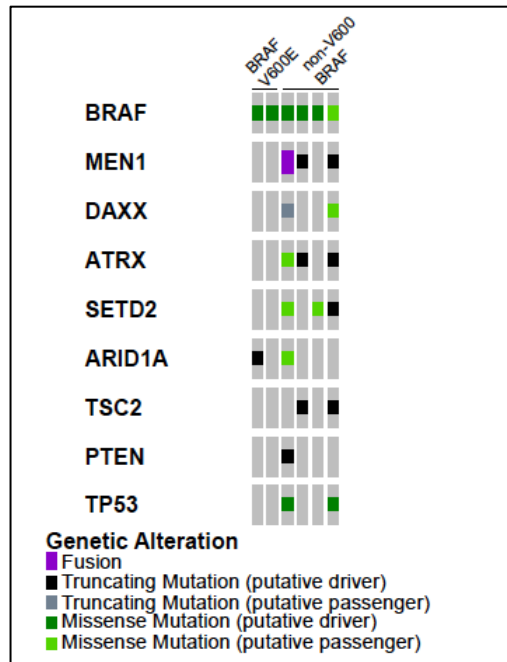

Supplement: S1 Fig — Series, (n = 6), shown to demonstrate overlap with genes from Raj et al. (MSKCC clinical series of panNET, [31]). (PDF) [file pone.0217399.s002.pdf]

S2 Figure

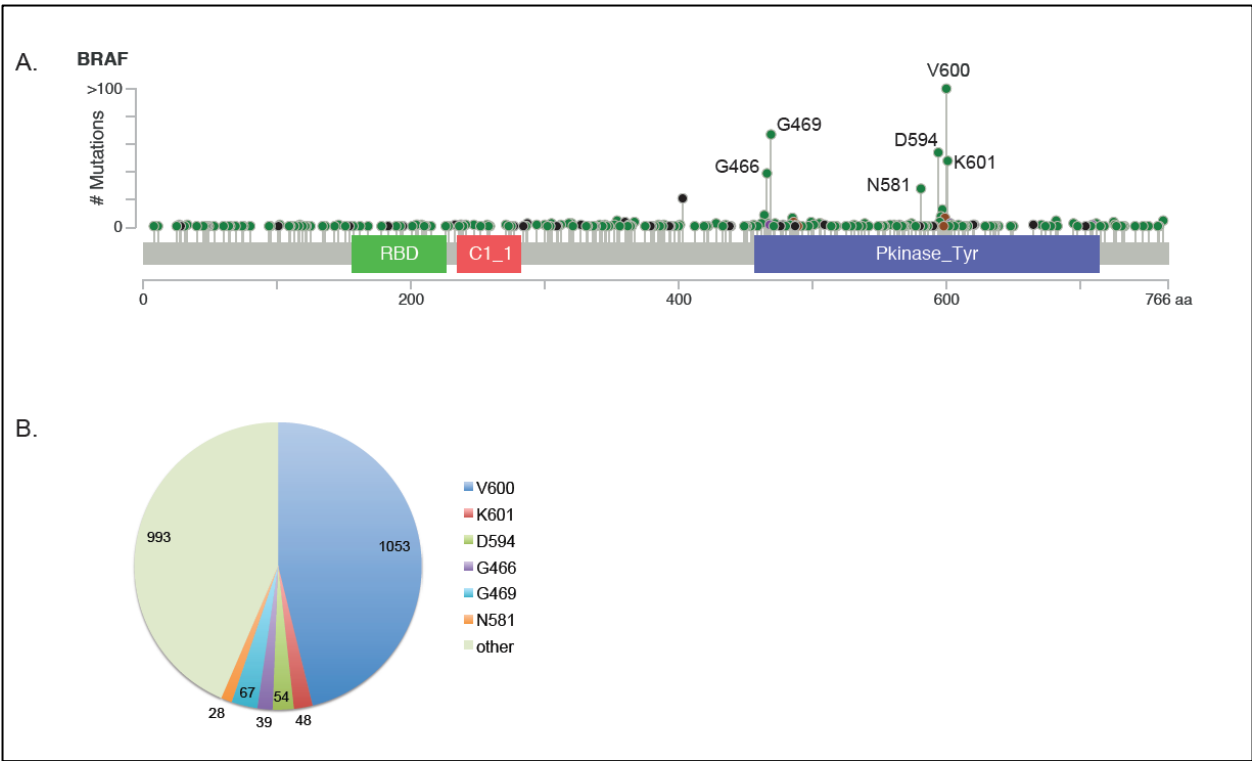

Supplement: S2 Fig — A. Lollipop plot of all missense, in-frame, truncating, and other mutations in BRAF, n = 2282; V600 represents the most commonly altered codon, and the y-axis is set at 100 to enhance visualization of next five most common codons altered in cancer. B. Distribution of V600, K601, D594, G466, G469 and N581 mutations among cases in which BRAF is altered (n = 2282). References [28–30]. (PDF) [file pone.0217399.s003.pdf]

S3 Figure

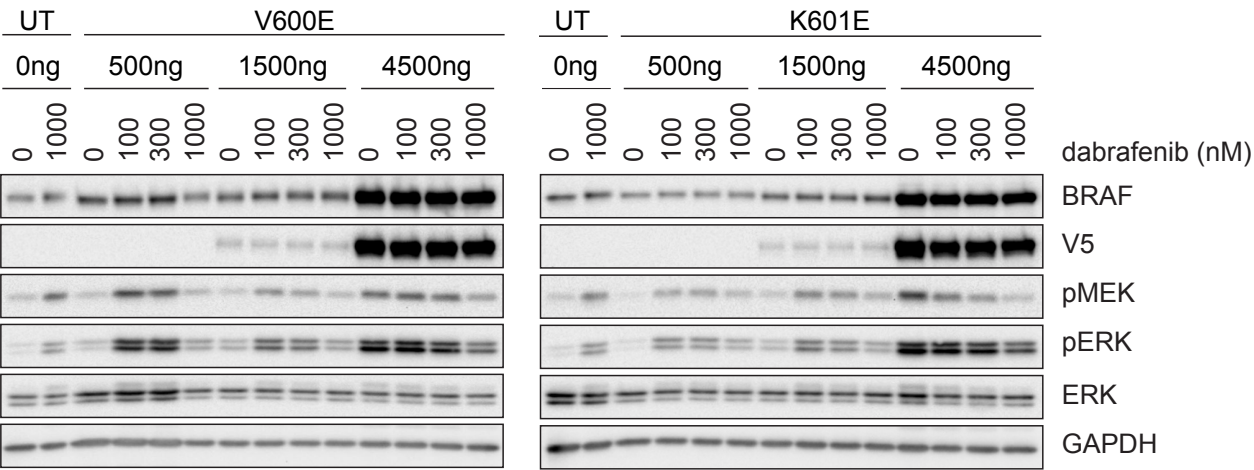

Supplement: S3 Fig — V5-tagged BRAF mutants (as indicated) were expressed in BON (NRAS Q61R) cells [32] overnight and followed by treatment with dabrafenib, over a dose range, for 1 hour. UT = untransfected. Expression and/or phosphorylation of the indicated proteins were assessed by immunoblot. (PDF) [file pone.0217399.s004.pdf]

## S4 Figure

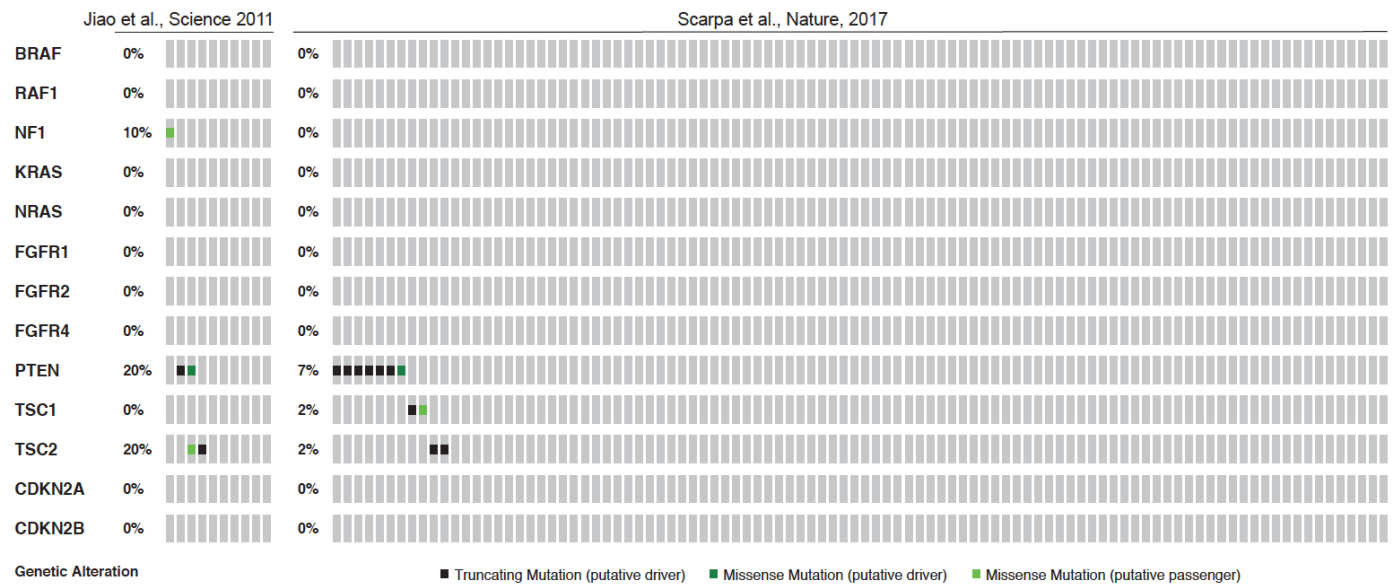

Supplement: S4 Fig — Genes encoding members of the RTK/ RAS/ RAF/ PI3K pathways in which mutations were found in our series (BRAF RAF1 NF1 KRAS NRAS FGFR1 FGFR2 FGFR4 PTEN TSC1 TSC2 CDKN2A CDKN2B, see Fig 1B) were used to query two published panNET data series using cBioPortal.org: Jiao et al, Science 2011, (BRAF mutations in 0 out of 10) [1]; and Scarpa et al, Nature 2017, (BRAF mutations in 0 of 98) [2]. (PDF) [file pone.0217399.s005.pdf]
