# Supplementary material for: Rare BRAF mutations in pancreatic neuroendocrine tumors may predict response to RAF and MEK inhibition
Source: PLoS One. 2019 Jun 3;14(6):e0217399. doi: 10.1371/journal.pone.0217399 (PMC6546234; doi:10.1371/journal.pone.0217399)
Supplement: S1 Table — Her 2013 tumor recurrence, and only one of her two resected recurrent sites of disease in 2014, harbored the BRAF K601E mutation. The other lymph node metastasis, resected, and from which the cell line was attempted, was confirmed to have no evidence of BRAF K601E (0 of 861 reads). (PDF) [file pone.0217399.s006.pdf]

S1 Table

source	specimen	total reads	K601E reads	VAF
2003 tumor	FFPE	260	1	0.0038
2003 tumor	Frozen	1334	1	0.0007
2004 tumor	FFPE	335	0	0.0000
2010 tumor	FFPE	803	1	0.0012
2013 tumor	FFPE	928	14	0.0151
2013 tumor	frozen	1372	110	0.0802
2014 tumor 1	FFPE	848	129	0.1521
2014 tumor 2	FFPE	861	0	0.0000
2014 tumor 2	cell line	1359	0	0.0000
